# School-based malaria prevalence: informative systematic surveillance measure to assess epidemiological impact of malaria control interventions in the Democratic Republic of the Congo

**DOI:** 10.1186/s12936-018-2297-2

**Published:** 2018-04-03

**Authors:** Edouard K. Swana, Thierry I. Yav, Leonard M. Ngwej, Betty N. Mupemba, Suprianto, Clarence K. Mukeng, Izak Hattingh, Oscar N. Luboya, Jean-Baptiste S. Kakoma, Michael J. Bangs

**Affiliations:** 1 China Molybdenum Company International, Ltd/International SOS, Public Health Programme, Tenke Fungurume Mining Project, Lualaba, Democratic Republic of the Congo; 2 Public Health Referral Laboratory, Lubumbashi, Haut Katanga Province Democratic Republic of the Congo; 3 Faculty of Medicine, University of Lubumbashi, Lubumbashi, Democratic Republic of the Congo; 4 School of Public Health, University of Lubumbashi, Lubumbashi, Democratic Republic of the Congo; 5 Public Health & Malaria Control, International SOS, P.T. Freeport Indonesia, Kuala Kencana, Papua 99920 Indonesia

**Keywords:** Malaria prevalence, School-based monitoring, Malaria control, Democratic Republic of the Congo

## Abstract

**Background:**

In southern Democratic Republic of the Congo, malaria transmission is stable with seasonal fluctuations. Different measurements can be used to monitor disease burden and estimate the performance of control programmes. Repeated school-based malaria prevalence surveys (SMPS) were conducted from 2007 to 2014 to generate up-to-date surveillance data and evaluate the impact of an integrated vector control programme.

**Methods:**

Biannual SMPS used a stratified, randomized and proportional sampling method. Schools were randomly selected from the entire pool of facilities within each Health Area (HA). Subsequently, school-children from 6 to 12 years of age were randomly selected in a proportional manner. Initial point-of-care malaria diagnosis was made using a rapid detection test. A matching stained blood film was later examined by expert microscopy and used in the final analysis. Data was stratified and analysed based on age, survey time and location.

**Results:**

The baseline SMPS (pre-control in 2007) prevalence was approximately 77%. From 2009 to 2014, 11,628 school-children were randomly screened. The mean age was 8.7 years with a near equal sex ratio. After exclusion, analysis of 10,493 students showed an overall malaria prevalence ratio of 1.92 in rural compared to urbanized areas. The distribution of *Plasmodium falciparum* malaria was significantly different between rural and urban HAs and between end of wet season and end of dry season surveys. The combined prevalence of single *P. falciparum*, *Plasmodium malariae* and *Plasmodium ovale* infections were 29.9, 1.8 and 0.3% of those examined, respectively. Only 1.8% were mixed *Plasmodium* species infections. From all microscopically detected infections (3545 of 10,493 samples examined), *P. falciparum* represented 88.5%, followed by *P. malariae* (5.4%) and *P. ovale* (0.8%). Cases with multiple species represented 5.3% of patent infections. Malaria prevalence was independent of age and gender. Control programme performance contributed to a significant decrease in mean *P. falciparum* infection density in urban compared to rural locations. Some rural areas remained highly refractory to control measures (insecticide-treated bed nets, periodic indoor residual spraying).

**Conclusion:**

The SMPS is a useful longitudinal measurement for estimating population malaria prevalence and demonstrating disease burden and impact of control interventions. SMPS can identify refractory areas of transmission and thus prioritize control strategies accordingly.

## Background

Throughout the Democratic Republic of the Congo (DRC), malaria is one of the leading health burdens in both rural and urban populations. In 2015, the DRC and Nigeria together accounted for more than 35% of the global total of estimated malaria deaths [1]. That same year, DRC contributed an estimated 12% of all malaria deaths in Africa [2]. Given the large percentage of the population living in high malaria endemic transmission areas, the country accounts for up to 11% of all *Plasmodium falciparum* cases in sub-Saharan Africa [3]. Countrywide, malaria is responsible for more than 40% of all outpatient medical visits and is the leading cause of reported morbidity and mortality, contributing an estimated 40% of deaths among children under 5 years of age [3]. These alarming statistics are likely an underestimation of the disease’s true overall health burden and demographic impact, especially in more remote areas that experience perennially high malaria incidence without, or at best, rudimentary control measures in place [4]. Unequivocally, malaria remains a major public health problem among school-aged children, its impact felt during this critical period of learning and development with likely long-term repercussions on society as a whole [5].

Direct government funding for malaria control is very limited and access to primary health care (public or private) remains scarce and problematic in many rural areas of the country. Current control activities and targets of the National Malaria Control Programme (NMCP) are primarily supported by bi- and multi-lateral international donors that focus on skill capacity building, health system strengthening, monitoring and evaluation, behaviour change and communication, malaria case management and the mass distribution of insecticide-treated bed nets [3]. Except for a few delimited areas in the country, the DRC is devoid of direct vector control activities such inter-domiciliary application of residual insecticides or control of vector mosquito larval habitats. Currently, indoor residual spray (IRS) is performed by only a few private companies involved in resource extraction (mineral mining).

Different measurement tools and methodologies have been used to monitor the burden of malaria in communities and estimate the impact of malaria control interventions for reduction of transmission [4]. These include passive clinical malaria case reporting by local public and private health services, parasite infection rates and *Plasmodium* species identification derived from point prevalence surveys or active case detection, and malaria vector-based metrics such as entomological indices (e.g., vector attack densities and number of infectious inoculations per person per unit time) [6–9]. Other diagnostic and research tools (e.g., serological markers, molecular-based methods) can provide greater detection sensitivity for operational control needs in different settings [10–13]. The restricted availability and accuracy of surveillance data in the DRC is hampered by a lack of reliable health services data, funding, basic training and access to more specialized and accurate measurement methods. However, the more basic, long-established tools for measuring essential malariological parameters (e.g., standard blood film examination) remain crucial and useful in normal and more remote operational settings.

Different approaches have been used to measure the parasite infection rate depending on objectives (research or operational) and availability of resources. Large-scale, population-based surveys targeting households might utilize standardized methodologies, such as ‘multiple indicators cluster surveys’, ‘demographic and health surveys’, and ‘malaria indicator surveys’ [14, 15]. More sophisticated, cluster-based surveys are limited as they are inherently expensive, time-consuming, and technically complicated to undertake. Sampling from smaller administrative units is typically powered to provide information relevant and extrapolative on a larger scale (e.g., provincial or national levels) to derive malaria infection estimates, relative transmission risk, and impact of interventions [16]. Conversely, school-based malaria prevalence surveys (SMPS) are typically far simpler to conduct and useful for more defined, local-level assessments, including routine longitudinal malaria surveillance. School surveys can provide both site-specific and period-time information on infection prevalence as a reliable indicator of malaria burden and transmission intensity in a defined community that can assist both health policy makers and operational control programme administrators with similar objectives [4, 16]. When well-organized and performed, the SMPS provides valuable information on intervention performance and for identifying priority areas requiring additional attention or different strategies [16–20]. Besides obtaining useful spatial and temporal data, together with the screening convenience of school-based children for sampling, the other inherent benefit is focusing greater emphasis and protection of this vulnerable age group [18].

**Fig. 1:**
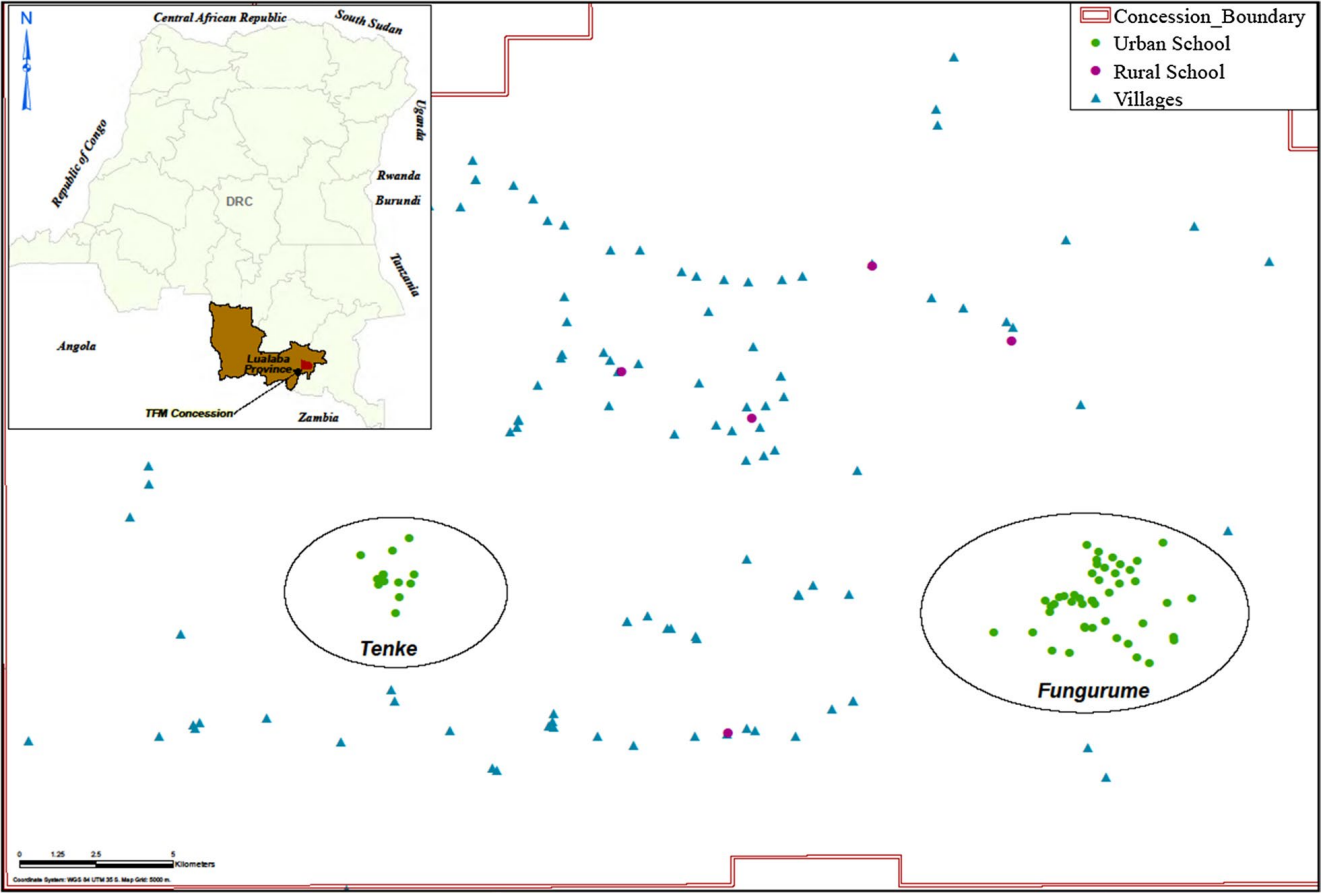
Inset showing location of Lualaba Province and TFM concession in the DRC. Larger map showing geo-referenced distribution of the two urbanized areas and rural villages with primary schools involved in the biannual malaria prevalence surveys

In 2007, Tenke Fungurume Mining (TFM), a private mineral resource company (an affiliate of Freeport McMoRan Inc.), located in Lualaba Province (formerly part of Katanga Province) in southern DRC (Fig. 1) began a major development operation for the large-scale commercial extraction of copper and cobalt ore. Concurrently, the company made an early and substantial social assistance commitment, in collaboration with national, provincial, and local health authorities, to reduce malaria morbidity and mortality in the resident mine operation workforce and surrounding mine-impacted communities. The extensive mine concession area consists of the townships of Tenke and Fungurume and numerous surrounding rural villages. Most communities in the urbanized areas have grown dramatically in size (both in population and infrastructure) since the beginning of mine development (2007) and production operations (2009). The number of primary schools has also seen a dramatic increase throughout the concession. From the initial design and implementation of a malaria and vector control programme (MVCP), the site-specific, integrated operation is currently responsible for protecting an estimated population of over 300,000 (Fungurume Health Zone census data 2016, unpublished). The mosquito vector control programme component has focused primarily on maintaining a highly organized, well-trained staff for providing routine IRS of community homes, the periodic mass distribution of long-lasting insecticide-treated bed nets (LLINs), and select locations for routine entomological monitoring and larval source management using environmental and/or larvicidal approaches as appropriate.

Herein are described the methodology used for the biannual SMPS and its temporal evolution to adjust to a rapidly growing population and dramatic changes due to economic development. The findings obtained in the 12 SMPS intervals covering 2009–2014 involving communities receiving malaria control interventions are presented.

## Methods

### Survey area

The surveys were conducted in the Tenke Fungurume Mining (TFM) concession. TFM is a private mineral resource company (actually an affiliate of China Molybdenum Company International, Ltd) located in the Fungurume Health Zone (FHZ) in the Lualaba Province (formerly part of Katanga Province) in southern DRC (Fig. 1).

In May 2006, an initial mosquito vector survey (Andreasen MH, unpublished report, Phelps Dodge Corp. USA) was conducted to identify the local malaria vectors and map predominant larval habitats. To date, at least 17 *Anopheles* species have been identified as potential malaria vectors in the operational coverage area. The primary (most predominant) year-round vector is *Anopheles gambiae* s.s., and secondarily *Anopheles coluzzii*, *Anopheles funestus* s.l. and *Anopheles arabiensis* are also present. The exact role of each species in malaria transmission during dry and wet seasons continues to be investigated. The latter two species are more often found in relatively low density throughout the year or seasonally in more focal locations. A malaria prevalence survey was not performed in 2006 but it was known that *P. falciparum* was the predominant (> 97%) malaria parasite detected from passive patent infections inside the FHZ, followed by *Plasmodium malariae* and *Plasmodium ovale*. In May 2007 (MJB, unpublished report), the first and only pre-control SMPS was conducted in 4 available schools in the FHZ concession area including 2 urbanized and 2 rural communities. This initial survey serves as the ‘baseline’ malaria prevalence rate for all subsequent school surveys and comparisons to assess impact of the control programme.

A community-wide IRS programme was launched in October 2008 beginning with use of synthetic pyrethroids and simultaneous with an initial mass distribution of LLINs. The first intervention period school malaria prevalence survey (SMPS) was conducted in May 2009. Subsequently, each succeeding year a SMPS has been conducted in May and October to monitor and evaluate the impact of the control programme. The information has provided timely feedback to the MVCP and Health Area (HA) regards operational progress and possible deficiencies in control for each respective area. Additionally, the malaria and vector control programme (MVCP) established early on a routine entomological surveillance programme to evaluate impact of interventions on the local vector populations and monitor any changes in insecticide susceptibility patterns over time. From inception, this control programme has been wholly sponsored by TFM with the operational responsibility provided by a contracted medical provider. FHZ is one of the 14 Ministry of Health administrative zones in the province with centralized government authority residing in the town of Kolwezi (Fig. 1). The FHZ is divided into 18 Health Areas (HAs) designated primarily based on population size in each. As of 2016, the MVCP has covered 12 HAs in the FHZ which has expanded dramatically in recent years to greater than 300,000 (est. 2016) residents.

In DRC, each HA consists of a set of rural villages and/or urbanized areas with an estimated population of around 10,000 inhabitants. Therefore, more densely populated areas (e.g., larger villages, towns, cities) would cover relatively smaller geographic areas compared to areas consisting of smaller, more widely dispersed villages. In general, an urban HA would also have a higher population number than a rural HA. Each HA may have several private or government-supported primary health care clinics but only one facility will generally serve as the assigned Health Centre responsible for the entire HA [21].

For purposes of survey collections and analysis, schools were divided by HAs into two broad designations, as either ‘rural’ or ‘urban’ (and more urbanized periphery), based on criteria of location and relative environmental/demographic conditions. In some instances, the separation between the two areas is ambiguous and represent a transitional phase (for example, ‘semi-urban’) between the two, i.e., a combination of attributes than define more clearly one or the other designations. Moreover, children attending schools in one HA may come from afar from another HA, thus not truly representing the malaria intensity nearer the school location.

In this setting, ‘urban’ is generally defined as having access to the public electricity grid (but not necessary connected or having uninterrupted power), the presence of commercial businesses, and with both paved, but mostly dirt/gravel roads. The vast majority of urban area residents also lack in-house piped water or dedicated sewage systems. Access to clean water is variable with a greater reliance on universal community-supplied ground water pumps. Housing varies from smaller traditional dwellings made of unbaked/baked clay (majority with metal roofing) to larger, more modern designed structures using alternative construction materials (e.g., plastered clay walls or cement block). Economic status is variable, from employment opportunities with commercial enterprises and local industry to farming. Conversely, rural areas have a much lower population density, lacking electricity and are comprised almost entirely of traditional structures with either metal or thatch roofing. Vehicular roads are limited and poorly maintained with access to some villages by footpath (bicycle/motorbike) only. Access to fresh water is variable, either from a community pump, natural well or surface water depending on the time of year. Formal employment is very limited with the vast majority of inhabitants involved in subsistence level seasonal farming.

Two distinct and near equal time periods representing the wet and dry season occur in the area. The general environment, landscape, climate suitability and socio-economic development is regarded as highly conducive for maintaining high levels of malaria infection and perennial transmission, albeit with some seasonal fluctuations [22–24]. The entire control coverage is a combination of 48 (as of end of 2014) small to medium-sized rural villages and two larger urbanized (‘urban’ towns) areas surrounded by varying expanses of native open dry forest ecology and agricultural plots. The Miombo woodlands ecosystem also contains a mix of natural woodland savannah and large tracts of human-disturbed areas due to deforestation, charcoal production and traditional agriculture. The so-called ‘savannization’ process in the region continues at a rapid pace, and combined with increasing demographic growth and human development expansion, contributes to the high malaria risk.

In October, with the beginning of the normal wet period, malaria transmission increases quickly during the following months with the typical peak in clinic-based reported cases occurring between December and March [MVCP unpublished reports]. Malaria cases remain elevated during and immediately following the period of heaviest rainfall (December–February) and during the gradual decrease of rains in March and April. Malaria transmission decreases further into the predominantly dry, cooler period (May–July) with reported infections reaching their lowest number during the warmest and driest months (August–September) of the year.

### School and student selection process

These are cross-sectional surveys repeated twice each year with school children randomly selected each round. A stratified, randomized, proportional sampling method of school children was organized in two stages. First, existing primary grade schools at time of survey were randomly selected from the entire pool of available facilities within each HA under MVCP coverage. No school declined participation if selected. Second, a random sampling from all identified eligible school children was performed within each selected school. The total number of children per school was determined in a proportional manner based on most current student enrollment data.

#### School selection

Primary schools (grades 1–6) were randomly selected within each HA. Those schools located near the perimeter of the IRS coverage zone were excluded as a larger percentage of attending children resided outside the control zone.

To begin, a list of all primary schools (government, private, parochial) and the current school enrollment register of each is provided by the local government Inspector of Education. In the 2007 baseline collections, all existing primary schools were included in the survey. As the number of schools increased each succeeding year, the inclusion of additional schools in each HA was combined to derive an average in the coverage area and subsequently used in the selection process. For example, if the average was five schools per HA, all schools within a HA that has five or less schools were included. For an HA with more than five schools, an online random number generated table was used to select only five schools to be surveyed [25].

#### Selection of students

Table 1 shows the sample sizes and schools sampled from 2009 to 2014. The collection strategy sought to define the prevalence of *Plasmodium* infection in each HA within 95% confidence limits, with 4% precision and design effect of 1. A non-response rate (students who decline or become unavailable to participate) of 10% was included in the sample size calculations. As the overall malaria prevalence began to decline in 2009 following intervention, the number of enrolled students (and schools) increased in number inside the coverage area, therefore influencing the number of students sampled over time [26–28].

**Table 1:** Summary malaria prevalence surveys during intervention period from 2009 to 2014: number of schools selected from total and sample size from registered student population

Survey period	Schools operating in survey area		Students registered in the survey area			Schools sampled			Students sampled		
	Total schools	Percentage of urban schools	Rural	Urban	Total	Rural	Urban	Total	Rural	Urban	Total
May-09	29	93	342	11,312	11,654	1	9	10	26	569	595
Oct-09	29	93	352	11,365	11,717	1	9	10	43	545	588
May-10	30	87	1203	11,977	13,180	2	16	18	39	689	728
Oct-10	38	89	1255	13,052	14,307	3	13	16	107	763	870
May-11	38	89	1319	16,927	18,246	4	20	24	132	879	1011
Oct-11	40	88	1364	16,801	18,165	5	19	24	128	971	1099
May-12	43	88	1428	18,475	19,903	5	20	25	117	979	1096
Oct-12^a^	48	90	1124	16,305	17,429	5	20	25	116	1019	1135
May-13	48	90	1597	18,604	20,201	5	20	25	143	967	1110
Oct-13	52	90	1540	17,782	19,322	5	20	25	145	918	1063
May-14	54	91	1448	19,775	21,223	5	20	25	150	1041	1191
Oct-14	59	92	1389	18,923	20,312	5	20	25	154	988	1142

^a^October 2012 survey excluded from final analysis as only RDT results are available

For each survey period, after calculating the total sample size required, the selected schools were assigned numerically with the most recent list of registered student population of each used to derive the proportion of samples each school would contribute to the total sample size under consideration. The same proportionality was used for calculating the final sample size for each school. All school children aged from 6 to 12 years old and present on day of blood sampling were eligible to participate in the survey. Children were not stratified by specific age (or age category) or gender. Each student received a card with a unique number. In addition to the selection number, the card provided space to record the student’s name, gender, primary classroom, and the axillary temperature at time of blood sampling. An online random number generated table [25] was used for the final selection of students based on the calculated minimum sample size per school. Sample selection was organized by the MVCP survey field supervisor and team in the presence of the respective school headmaster and area Inspector of Education.

Each school was provided with a specific alpha-numeric code that identified the HA where the school was located, followed by a sequential number for each child selected and placed on the sample list. Thus, if 25 schools were selected, schools were coded per HA and numbered from 01 to 25. As example, the first student from the first school coded, ‘DA01’ would have the code DA0101, while the 35th student from the 5th school coded DB05 would be coded DB0535.

### Inclusion/exclusion criteria

The survey inclusion criteria were: (i) all school-children aged from 6 to 12 years of age present at school the day of the survey, (ii) willingness (as volunteers) to take part in all aspects of the survey and follow-up, and (iii) primary residence (i.e., majority of time spent) being within the MVCP intervention zone. Students were excluded from the survey if (i) under the age of 6 or above 12 years of age, (ii) primary residence outside the intervention zone, (iii) unwilling to participate or specific parent/guardian disapproval (‘opt-out’) after being informed about the survey, its intent and benefits, and (iv) sharing the same bed with a fellow student (potential bias in infection risk). Outright refusals to participate were very low and there were no negative repercussions for any child (or parent/guardian/legal representative) refusing to participate.

### Blood survey procedures

Selected participants, all as willing volunteers, were aggregated in a separate classroom specifically for information processing and blood testing. For each student called, the axillary temperature was measured using a digital thermometer (Eco Temp Basic MC-246-E, Omron Health Care Co, Ltd, Kyoto, Japan) and recorded on a paper form to include the following information: name of student, age, gender, class level and any history of having taken malaria treatment in the previous 2 weeks. In some survey periods, additional questions were asked about house spraying, bed net retention and utilization. In a few surveys, haemoglobin levels were estimated by using a field-compatible HemoCue^®^ Hb 201^+^ analyzer (HemoCue AB, Ängelholm, Sweden). Data on haemoglobin and other parameters are not provided herein.

Commercially available, single use malaria immunochromatographic rapid detection tests (RDT) was used for diagnosis. Children were asked to provide a small sample of finger-prick blood (approximately 5 µl) to test for evidence of *Plasmodium* parasites in the peripheral blood. RDT procedures strictly adhered to product instructions. A matching thick and thin blood film was made at the same time. Giemsa-stained blood slides were examined later for RDT diagnostic confirmation and assessment of RDT product performance (findings to be reported elsewhere). RDT cassettes and matching blood slides were provided an identical numbered code linked to the field record form. RDT results were obtained within 15 min of blood sampling. In compliance with DRC policy for restricting anti-malarial treatment to only those cases with parasitological-confirmed infections, children with malaria reactive (‘positive’) RDT results were immediately provided an approved, fixed combination, artemisinin-based combination treatment (ACT).

Thick and thin Giemsa-stained blood slides were subsequently sent to an external laboratory for examination by a certified expert using standard light microscopy. The examiner was blinded to the matching RDT results. For cost and timing reasons, there was no second independent examination of the blood films by another expert microscopist for verification or to reconcile discrepancies between initial RDT and slide findings. Blood slide examination was done using a minimum of 200 oil-immersion high magnification fields (1000×) before reporting a sample as parasite ‘negative’. Asexual and sexual parasite densities of all *Plasmodium* species seen were reported separately per 200 white blood cells. The total asexual parasites of *P. falciparum* per µl of blood was estimated using the conventional conversion factor of 40× (with assumption of approximately 8000 WBC present per µl normal whole blood).

Each blood slide result was compared to the matched RDT. The microscopic examination results were deemed definitive and were used in the final analysis. However, in some cases when there was a discrepancy (e.g., apparent ‘false-positive’ RDT), a quantitative real-time PCR method was performed (findings not reported herein). This was done on an ad hoc basis for quality assurance and did not serve any purpose in the analysis. Malaria prevalence results were compared to previous SMPS to view longitudinal changes in prevalence between years, influence of season, differences within schools, within and between each HA, parasite species frequency and infection densities.

### SMPS quality assurance

A school survey guideline was developed for each survey period and appropriate initial training or refresher training provided the day before commencing collections. Two teams (six persons each) were deployed to designated schools previously selected to participate. Each team was composed of two field ‘mobilizers’ responsible for sample selection and recording essential information from selected students; one nurse to perform axillary temperature and provide treatment for all RDT positive malaria cases; and two laboratory technicians for the blood sample collection for RDT and blood films. All blood slides were returned to the clinical laboratory and stained with Giemsa the same day. One field supervisor accompanied each team for managing the entire process in addition to a senior laboratory technician. The supervisor reviewed each completed form to ensure all information was recorded per child. These same forms were subsequently reviewed and approved by the primary investigator. If any errors or ambiguity were found at any stage in the process, appropriate remediation steps were undertaken and corrections made before final acceptance.

All data was entered to Epi Info™ version 5 (CDC, Atlanta, USA) the same or immediate following day of survey by a dedicated data management person. Any inconsistencies discovered at the time of data entry, the form was returned immediately to the field team for clarification. The primary investigator conducted random field inspections and data audits throughout the survey period.

### Ethical review

Ethical clearance was obtained from the Medical Ethical Committee of the University of Lubumbashi. All survey participants were volunteers. As all procedures, including blood sampling, were deemed of minimal risk, therefore only a verbal informed consent was obtained following standard procedures approved by local government authorities. General information and answers to frequently asked questions were sent to each headmaster of a selected school to convey information to those teachers to be involved in the survey. In turn, school administrators were responsible for informing all parents and allowing them to verbally opt-out (decline consent) for their children to take part in the survey. In the event of accidental exposure (e.g., inadvertent lancet stick), HIV counseling and post-exposure prophylaxis for HIV infection were available. As an added precaution, laboratory technicians performing blood sampling were pre-immunized against Hepatitis B virus.

### Analysis

Raw data was exported from Epi Info™ to Excel 2007 (Microsoft Corporation, Redmond, WA) for analysis using SPSS version 21.0 (SPSS Inc., Chicago, IL). Means (± standard deviation) and proportions were produced for all categorical variables. Malaria infection prevalence (based on observed patent parasitaemia) was calculated as overall prevalence and separately between rural and urban HAs and season (time of the survey). Confidence intervals (95% CI) were estimated around point estimates. Statistical inference was performed using Pearson Chi square test (with or without Fisher’s exact test, when applicable). The strength of association between parameters were estimated by a prevalence ratio with 95% CI [29]. The homogeneity of the distribution of malaria cases between rural and urban areas and by seasonality was estimated using the Breslow-Day test for homogeneity (i.e., relationship between exposure and outcome is either different or same for different strata).

Parasite densities were analysed for *P. falciparum* infections only, ranging from the minimum of 1 asexual parasite per 200 WBCs (≤ 40 parasites/µl blood) and higher. Infections presenting only gametocyte stages were excluded as active infections. Because of the high right asymmetry of the distribution of *P. falciparum* densities, a logarithmic (log_10_) transformation of *P. falciparum* density was used before analysis. The association between age groups (6–8; 9–10 and 11–12 years) and transformed *P. falciparum* density groupings (< 2, 2–3, and > 3) was analysed using the Pearson Chi square statistic. Spearman rho was used to assess correlations between *P. falciparum* densities over time (between surveys). Unless otherwise stated, all statistical significance was set at 5% (p < 0.05).

Figures with 95% interval error bars were generated using the STATISTICA software (v 1984-2011, StatSoft. Inc, Tulsa, USA). Where blood film examination results were not available, the percent prevalence is an approximate measure based on initial RDT results and adjusted using a percent error statistic derived from the difference between matching RDT and blood films results conducted in the preceding school survey. A regression log-binomial model was used to assess the association between survey year and infection.

## Results

### Schools and students sampled

During intervention from May 2009 to October 2014, a total of 43 primary schools (37 in urbanized and six in rural areas) located across 8 HAs under routine IRS and LLIN coverage were surveyed. All selected schools each period participated fully and compliance throughout was excellent. In all, 11,628 students were randomly identified for sample inclusion over the 12 survey periods. Approximately 89% of sampled students were residing in the designated urbanized zones based on corresponding HA and matching closely to the 88% of the total population living inside the TFM concession. The ages of sampled students ranged from 6 to 12 years (mean 8.7 SD ± 2.1) with an approximate 1:1 sex ratio (5962 males and 5666 females). Unfortunately, the October 2012 survey blood slides (n = 1135) were lost during commercial transport from DRC to the reference laboratory, thereby precluding expert microscopy. Therefore, the final analysis is based on 10,493 examined blood slides equivalent to the 11 relevant survey periods.

The total number of public and private primary schools available for selection and those selected increased each succeeding year. In May 2009 (first survey), 29 schools were active in the coverage area, of which 10 (35.5%) were randomly selected (Table 1). In October 2014 (last survey analysed), the school number had increased to 59, a 103% increase from 2009, of which 25 (42%) were sampled. This 150% increase in sampled schools over a 6-year period resulted in a commensurate increase in workload to perform the surveys within the targeted 14 consecutive working days. The total proportion of students randomly selected remained very similar between years (5.6–6.0%), but resulted in an increase in samples overall. From 2009 to 2014, the mean annual number of blood films increased from 592 to 1166, a 97% increase in workload. In that same time span, the number of schools increased over twofold in the urbanized areas and near periphery compared to rural areas, whereas the number of registered students (i.e., better access to schools) increased more dramatically (fourfold increase) compared to 70.6% rise in enrollment in urban schools. Nevertheless, the ratio of schools present between urban and rural areas remained constant (10:1). This information also provided a strong surrogate measure of the rapid population growth and area expansion in the FHZ as a direct result of greater economic activity promoted by the mine.

### Malaria prevalence

Results from the pre-intervention baseline MSPS conducted in May 2007 revealed a malaria point parasite prevalence of 77.05% (4 schools, n = 536). Sampling was based on convenience only (all those attending that day) for children aged 6–12 years old. Point-of-care diagnosis was made using an RDT with a matching blood film. Infection prevalence by school ranged from 66.6 to 88.9%. *Plasmodium falciparum* was the predominant parasite species among those observed infected on blood film, either as single or mixed species infections, ranging from 93.3 to 99%. The other two species seen, *P. malariae* and *P. ovale*, were predominately co-infections (63.6–94.7%) with *P. falciparum*.

**Fig. 2:**
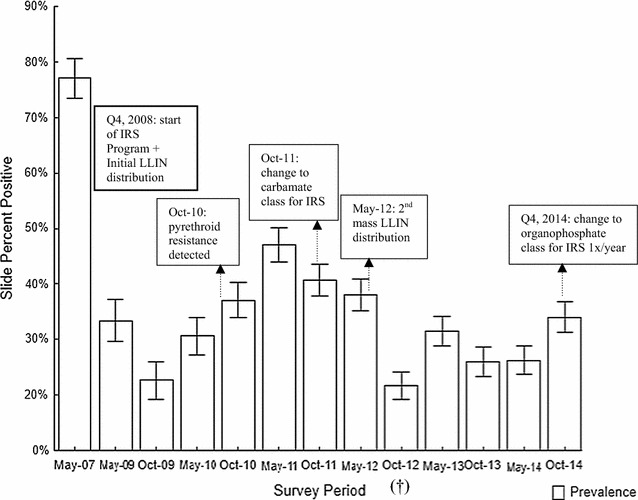
School-based malaria prevalence based on blood film examinations from 2007 to 2014 and timeline of key intervention and decision points in community malaria control campaign. ^†^October 2012 blood films were not available for examination. Therefore, the percent prevalence is an approximate measure based on initial October RDT results and adjusted using a percent error statistic (0.329) derived from the difference between matching RDT and blood films results conducted in the preceding May 2012 school survey

The malaria parasite prevalence findings during intervention (May 2009 to October 2014) are presented in Fig. 2. In May 2009 (approximately 6 months after beginning IRS/LLIN intervention), the overall prevalence decreased significantly (p < 0.001) from 77.05 to 33.8%. The malaria prevalence remained similar between May 2009 and May 2010 collections (p = 0.479) representing end of wet season transmission. During that same period an entomology laboratory with insectarium was set up to monitor local vector mosquito populations for insecticide susceptibility to the residual chemicals used for IRS inside the concession. From October 2010 to May 2011, the percent malaria cases increased significantly (p < 0.001) from approximately 37–46.9%, respectively. Vector susceptibility findings revealed a significant increase in resistance to pyrethroid class chemicals, which resulted in a change to a carbamate class formulation during the next round of IRS. Subsequently, the overall prevalence has not exceeded the May 2011 rate and reached its lowest estimated point (21.66%) in October 2012.

### Malaria prevalence, location, and time of the survey

Between rural and urban areas, the overall malaria prevalence ratio (PR) was nearly two times (1.924, 95% CI 1.818–2.036, p < 0.0001) greater in rural compared to urban areas (Table 2). However, there was no significant association (seasonal effects) between the time of survey (May and October) and malaria prevalence ratio [PR = 1.049, 95% CI 0.994–1.107, p = 0.083]. When stratifying between rural and urban localities, the risk of being infected in a rural area was 1.139 (95% CI 1.035–1.254, p = 0.007) and was more likely at the end of the wet season (May) compared to end of the dry season (October). In urbanized localities, there was no significant association between the biannual surveys [PR = 1.044 (95% CI 0.982–1.111), p = 0.17].

**Table 2:** Malaria prevalence ratio by location (urban or rural) and time of the survey (May or October)

Survey period and location (U = urban/R = rural)				Blood film results		Samples	Prevalence ratio* (CI: 95%)
				Positive	Negative		
May		R	Number	380	227	607	2.007 (1.865–2.161)
			%	62.6	37.4		
		U	Number	1598	3526	5124	
			%	31.2	68.8		
	Total		Number	1978	3753	5731	
			%	34.5	65.5		
October		R	Number	317	260	577	1.839 (1.686–2.007)
			%	54.9	45.1		
		U	Number	1250	2935	4185	
			%	29.9	70.1		
	Total		Number	1567	3195	4762	
			%	32.9	67.1		
Total		R	Number	697	487	1184	1.924 (1.818–2.036)
			%	58.9	41.1		
		U	Number	2848	6461	9309	
			%	30.6	69.4		
	Total		Number	3545	6948	10,493	
			%	33.8	66.2		

* All Chi square comparisons between locations were highly significant (p < 0.0001)

When stratifying by seasonal period (based on time of the survey), the May findings showed a significant association between malaria prevalence in rural compared to urban areas [PR = 2.007 (95% CI 1.865–2.161), p < 0.0001]. The October results, more reflective dry period effects showed that risk of being infected was 1.839 [(95% CI 1.686–2.007), p < 0.0001] greater in rural compared to urbanized localities. Thus, malaria prevalence was higher in rural localities with minimal influence of time of year and prevailing seasonal conditions.

**Fig. 3:**
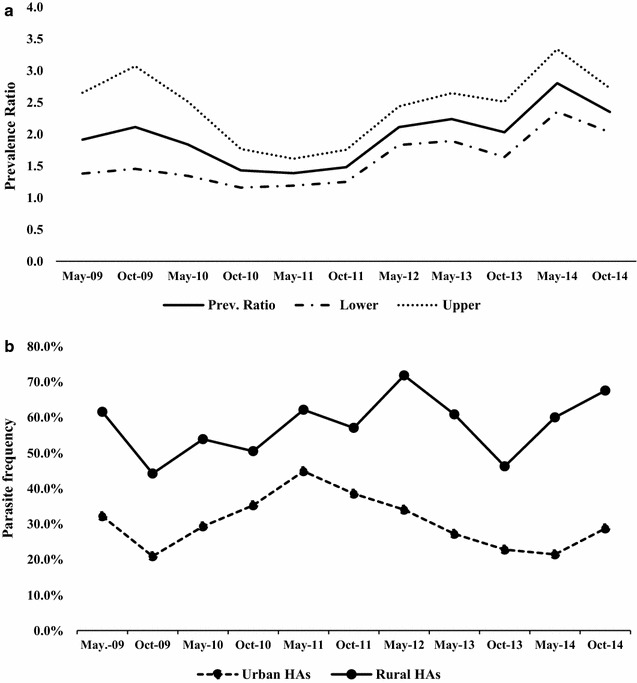
Estimation of malaria risk and infection frequency between rural and urban Health Areas (HAs). **a** Estimation of mean malaria risk with upper and lower range in rural vs. urban HAs from 2009 to 2014. **b** Parasite infection frequency (all species) in urban and rural HAs from 2009 to 2014

Tests for homogeneity between location (rural or urban HA) and time (May or October) found the distribution of malaria cases was different between rural and urban HAs and between May and October surveys (Breslow-Day Chi square, p = 0.044), indicating presence of an interaction between survey time and location. Figure 3a, b presents the estimation of malaria risk (PR) and malaria parasite frequency in urban vs. rural HAs, respectively. In sampled rural schools, the malaria prevalence never went below 40% and was always between 1.5 and 3.0 times high in rural compared to urban HAs. There was a notable spike in malaria parasite prevalence in May 2012 in rural areas while there was a decrease during the same period in urban localities.

### *Plasmodium* species

**Fig. 4:**
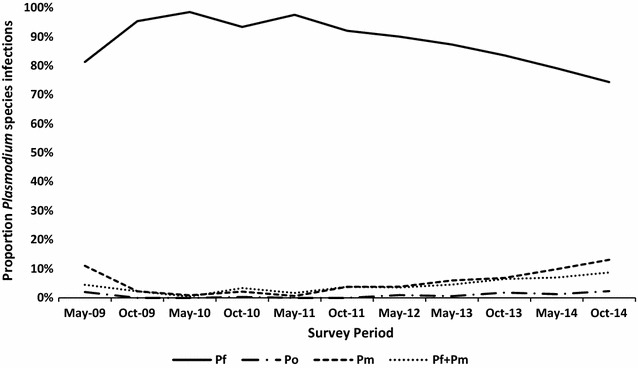
Proportion by *Plasmodium* species detected by blood slide examination from 2009 to 2014

From a total of 10,493 slides examined, 3545 (33.8%) were found malaria positive for one or more *Plasmodium* species, covering 11 survey periods during a 6-year intervention phase. The overall prevalence of single infections with *P. falciparum*, *P. malariae* and *P. ovale* were 29.9, 1.8 and 0.3% respectively. *Plasmodium vivax* was not detected morphologically or using real-time qPCR from a random sampling of *P. ovale* infections. Multiple species infections (all combinations) represented 1.8% of all malaria cases, wherein 1.5% were *P. falciparum* + *P. malariae*. Infections with all 3 species were also detected. *Plasmodium* species prevalence by survey period and relative proportions are shown in Table 3 and Fig. 4. From all malaria cases detected (3545), *P. falciparum* alone represented 88.5% of infections, followed by *P. malariae* (5.4%) and *P. ovale* in 0.8%. Overall, multiple species infections (all combinations) represented 5.3% of infections detected. Over time, the proportion of *P. falciparum* declined from a peak of 98.7% (May 2010) of infections to 74.5% (October 2014).

**Table 3:** *Plasmodium* species prevalence proportion (as percent) by survey period during intervention period 2009–2014

	May-09	Oct-09	May-10	Oct-10	May-11	Oct-11	May-12	May-13	Oct-13	May-14	Oct-14	Total
*P. falciparum* (Pf)	81.4%	95.5%	98.7%	93.5%	97.7%	92.2%	90.2%	87.4%	83.7%	79.2%	74.5%	88.5%
*P. ovale* (Po)	2.0%	0.0%	0.0%	0.3%	0.0%	0.0%	1.0%	0.6%	1.8%	1.3%	2.3%	0.8%
*P. malariae* (Pm)	11.1%	2.2%	0.9%	2.2%	0.6%	3.8%	3.8%	6.0%	6.9%	9.9%	13.1%	5.4%
Pf + Pm	4.5%	2.3%	0.4%	3.4%	1.7%	3.8%	3.6%	4.6%	6.5%	7.0%	8.8%	4.3%
Pf + Po	1.0%	0.0%	0.0%	0.6%	0.0%	0.2%	1.0%	1.1%	0.7%	1.6%	0.8%	0.7%
Pf + Po + Pm	0.0%	0.0%	0.0%	0.0%	0.0%	0.0%	0.5%	0.3%	0.4%	1.0%	0.5%	0.3%
Total positive	199	133	223	323	476	447	417	350	276	313	388	3545
Sample size (n)	595	588	728	870	1011	1099	1096	1110	1063	1191	1142	10,493
Prevalence (%)	33.4	22.6	30.6	37.1	47.1	40.7	38.0	31.5	26.09	26.3	34.0	33.8

### Malaria and body temperature

From the 10,493 apparently healthy children attending school the day of the survey, 366 did not have an axillary temperature recorded. From the 10,127 children that did, temperature at time of blood sample ranged from 35.1 to 39.9 °C (mean 36.7 °C SD ± 0.43). Only 325 students (3.2%) had a defined ‘fever’ (elevated temp ≥ 37.5 °C) (Table 4). From those who had fever, 41.8% (136) had a detectable malaria infection. There was a significant association between having a fever and malaria infection (p = 0.002). Students with fever on survey day were 1.3 times [95% CI 1.1–1.4] more likely to be infected with malaria compared to those without fever.

**Table 4:** Malaria infection and body temperature at time of blood sampling by period of year and location

Strata		Presence of fever (≥ 37.5 ℃)	Blood smear results		Total	p-value*	Prevalence ratio (CI 95%)
			Positive (%)	Negative (%)			
Period	May	Yes	61 (43.0)	81 (57.0)	142	0.029	1.259 (1.038–1.527)
		No	1782 (34.1)	3441 (65.9)	5223		
	October	Yes	75 (41.0)	108 (59.0)	183	0.018	1.258 (1.052–1.504)
		No	1492 (32.6)	3087 (67.4)	4579		
Location	Rural	Yes	28 (70.0)	12 (30.0)	40	0.144	1.198 (0.972–1.476)
		No	659 (58.4)	469 (41.6)	1128		
	Urban	Yes	108 (37.9)	177 (62.1)	285	0.005	1.257 (1.080–1.463)
		No	2615 (30.1)	6059 (69.9)	8674		
Total		Yes	136 (41.8)	189 (58.2)	325	0.002	1.253 (1.099–1.428)
		No	3274 (33.4)	6528 (66.6)	9802		
		Total	3410	6717	10,127		

When data were aggregated by time of survey, both May and October periods showed a significant association between fever and presence of malaria infection. In May and October, the PR’s were equivalent, 1.259 [95% CI 1.038–1.527, p = 0.029] and 1.258 [95% CI 95% 1.052–1.504, p = 0.018], respectively. However, when aggregating data by survey location (urban or rural), there was a significant association between fever and malaria infection in urban localities (p = 0.005) with PR 1.257 (95% CI 1.080–1.463), while showing no relationship (p = 0.144) in rural areas [PR = 1.198 (95% CI 0.972–1.476)]. Fevers due to other possible causes were not investigated.

**Fig. 5:**
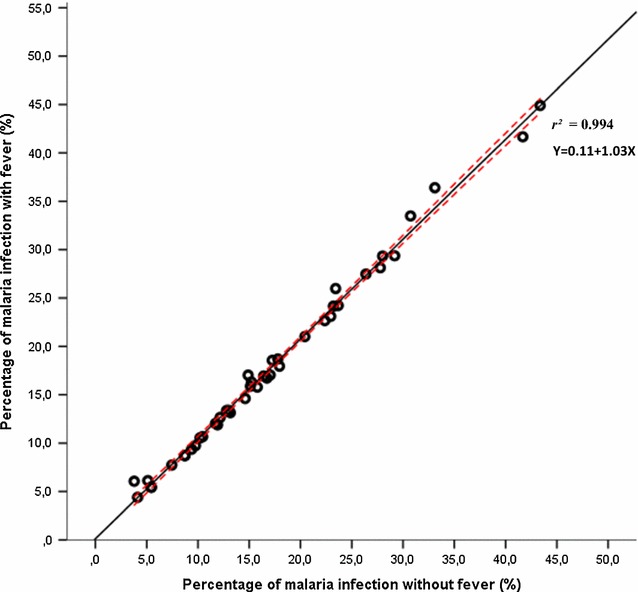
Coefficient of Determination (strength of the linear association) between malaria infection with fever and malaria infection without fever among 10,127 students among the 43 schools sampled from 2009 to 2014

Figure 5 shows the strength of the linear correlation between malaria infection with fever and malaria infection without fever among 10,127 students from the 43 schools sampled from 2009 to 2014 (95% CI Pearson Coefficient of Determination *r*^2^ = 0.994 with p = 0.001). These findings indicate that there is a positive correlation and a significant difference between infection in the presence or absence of fever.

### Malaria, age, and gender

All sampled children were aged from 6 to 12 years old. Three age groups were analysed: 6–8 years (n = 5276), 9–10 years (n = 2851) and 11–12 years of age (n = 2366). From 2009 to 2014, overall malaria prevalence in these groups were 32.8, 35.4 and 34.0%, respectively. Malaria infection was determined to be independent of age. There was no significant association between malaria infection and age groups (Chi square = 5.595, p = 0.061) indicating no difference in risk of exposure and transmission. Overall, there was no difference in malaria prevalence by gender (p = 0.0847), although there were slightly more males (54.7%) found infected.

### Parasite density by location and time

Association between *P. falciparum* density and age groups was analysed by location (urban vs. rural) and the survey period (Table 5). Based on location, there was a significant association between *P. falciparum* density and the 3 different age groups (p = 0.039). When stratified by location, there was no significant association between *P. falciparum* density and age groups in urban (p = 0.134) or rural areas (p = 0.138). However, a significant association between *P. falciparum* density and age groups (p = 0.039) was seen with time of survey. When stratified by time, May revealed no significant association (p = 0.648) with parasite density, while the end of dry season October surveys showed a strong association (p < 0.0001).

**Table 5:** *Pf* density by location, age group and surveys period

Location and age (years)			log_10_ *Pf* density			Total	p-value
			< 2.00	2.00–2.99	3.00+		
*Pf* density-aggregation by survey location							
Rural	Age	6–8	101	128	84	313	0.138
		9–10	46	70	39	155	
		11–12	59	70	26	155	
	Total		206	268	149	623	
Urban	Age	6–8	496	438	246	1180	0.134
		9–10	313	259	121	693	
		11–12	255	188	93	536	
	Total		1064	885	460	2409	
Total	Age	6–8	597	566	330	1493	0.039
		9–10	359	329	160	848	
		11–12	314	258	119	691	
	Total		1270	1153	609	3032	

Survey period and age (years)			log_10_ *Pf* density			Total	p-value
			< 2.00	2.00–2.99	3.00+		
*Pf* density-aggregation by survey period							
May	Age	6–8	281	311	182	774	0.648
		9–10	172	206	112	490	
		11–12	168	163	92	423	
	Total		621	680	386	1687	
October	Age	6–8	316	255	148	719	0.000
		9–10	187	123	48	358	
		11–12	146	95	27	268	
	Total		649	473	223	1345	
Total	Age	6–8	597	566	330	1493	0.039
		9–10	359	329	160	848	
		11–12	314	258	119	691	
	Total		1270	1153	609	3032	

The correlation between log_10_ *P. falciparum* densities throughout the intervention period (2009–2014) was examined for urban and rural HAs. Overall, there was an inverse correlation (Spearman rho) between the time of the survey and *P. falciparum* densities (n = 3032, significantly different to zero *r *= − 0.122, p < 0.0001). In rural localities, the correlation was not different to zero (n = 623, *r *= − 0.069, p = 0.084), therefore, parasite density appeared independent with time. In urban localities, however the correlation was significantly different to zero (n = 2409, *r* = − 0.155, p < 0.0001), indicting a clear difference over time (i.e., a control effect) with *Pf* density decreasing in urban localities. By using the multivariate regression log-binomial model, there was an association between survey year and prevalence over the 6-year observation period (Table 6).

**Table 6:** Adjusted prevalence ratios using log-binomial regression in a context of a generalized linear model

Parameter	Hypothetical test			Exp (B)	95% Wald confidence interval for Exp (B)	
	Wald Chi-square	df	p value		Lower	Upper
(Constante)	916.569	1	0.000	0.123	0.108	0.141
Rural	551.337	1	0.000	2.961	2.705	3.242
Urban				1		
May	123.004	1	0.000	1.536	1.423	1.656
October				1		
Male	46.229	1	0.000	1.296	1.203	1.397
Female				1		
6–8 years of age	0.275	1	0.600	0.975	0.888	
9–10 years of age	2.076	1	0.150	1.080	0.973	1.198
11–12 years of age				1		
Presence of fever (≥ 37.5 °C)	7.811	1	0.005	1.243	1.067	1.447
Absence of fever (< 37.5 °C)				1		
Survey year 2009	13.725	1	0.000	1.354	1.153	1.589
Survey year 2010	76.404	1	0.000	1.778	1.563	2.023
Survey year 2011	128.499	1	0.000	1.980	1.759	2.228
Survey year 2012	8.083	1	0.004	1.204	1.059	1.368
Survey year 2013	6.316	1	0.012	0.838	0.731	0.962
Survey year 2014				1		
(Scale)						

Dependent variable: positive result

Model: (Constante), month, location, gender, age, temperature

Where Exp (B) is the PR estimate of a given covariate

## Discussion

Within the TFM concession, SMPS was used as the primary measurement tool for establishing a pre-control baseline malaria prevalence rate and subsequently the basis and means of quantitatively assessing the impact of the MVCP interventions over time. For intervention period surveys, schools were randomly selected in a proportional manner per HA to more accuracy represent the prevalence of malaria in the various coverage areas (urbanized and rural) inside the FHZ. By providing comparable longitudinal data from a reliable and informative population cohort, this evidence has been used to assist in the timely review and, when necessary, the reorientation in control programme policy and operational activities.

Standardized prevalence surveys were found useful as an objective, evidence-based tool for detection of increased malaria activity that was associated with the rapid development of insecticide resistance in the primary *Anopheles* vector mosquito population to the pyrethroid class compounds being used for indoor residual spraying (IRS) and factory-treated bed nets. This lead to the use of an alternative class compound (carbamate) for IRS (Fig. 2). Routine SMPS detected a more persistent high malaria prevalence in many rural health areas compared to more urbanized locations, indicating that additional or more intense interventions are necessary to reduce malaria burden in these more at-risk communities. Historically, longitudinal information of this type is very rare in the DRC, especially in areas far removed from the Kinshasa area where our control programme activities take place [30–34].

The baseline SMPS in 2007 confirmed that malaria was the leading cause of morbidity in the FHZ. Soon afterwards, a MVCP was set up with IRS as the main pillar for vector control in the community. Following the initial rounds of house-to-house indoor spraying, the mean malaria prevalence decreased significantly from above 77 to 22.6% by October 2009. Afterwards the biannual prevalence has varied from a wet season high of 47% (May 2011) to a dry season low of 21.7%. The prevalence at the end of this intervention reporting interval (October 2014) was 33.5%, an overall 55.8% reduction in malaria since control programme inception.

Following the introduction of broad coverage vector control measures, the malaria prevalence decreased rapidly. This result that can be attributed to the first-time introduction of pyrethroid-based IRS in the majority (> 90%) of households combined with mass distribution commercial long-lasting insecticide-treated nets. The pyrethroid active ingredients used (deltamethrin, lambda-cyhalothrin, and alpha-cypermethrin, as either wettable powder or granule formulations) provided +an approximate effective residual life of between 4 and 6 months depending on the type of surface sprayed. Initially, the single annual IRS spray round began just before the rains resumed with the intention of providing protection during the higher malaria transmission period (October to March) of the year. The survey strategy was based on the expectation for natural reduction in transmission between May and October attributed to negative effects on the local vector population during the prolonged dry period.

The initial steep reduction was seen up to the October 2010 (22.6%) survey, approximately 2 years following IRS introduction. However, from October 2010 onwards, the prevalence increased significantly (*p *< 0.001) from 30.6% up to 47% in May 2011. Beginning in May 2010, the MVCP entomology laboratory and insectarium was established and two insecticide susceptibility bioassays were performed: (1) the standard WHO tube contact test using wild-caught larvae reared to the female adults and exposed to insecticide-treated filter-paper according to protocol [35] and (2) field-based cone bioassays [36] on treated walls of homes to determine the sensitivity and longevity of insecticides on various types of sprayed surfaces against both a colonized insecticide-susceptible strain of *An. arabiensis* and wild-caught anophelines, primarily *An. gambiae* s.s. ‘S’ molecular form. Results from 2010 susceptibility assays found conclusive evidence of substantial levels of resistance in *An. gambiae* to pyrethroids (permethrin and deltamethrin) including cross-resistance against DDT. These tests confirmed that in a period of 2 years from inception of wide scale IRS and LLINs (both using pyrethroids), selection pressure was sufficient to produce operationally relevant resistance (> 20%) against pyrethroids. Thus, in 2011, a change was made from pyrethroid to a carbamate-based insecticide (bendiocarb). As carbamates typically have a shorter residual life of only 3–4 months on most sprayed surfaces, two spray rounds were required to cover the entire high malaria transmission period. In May 2012, a second mass distribution of LLINs was conducted. Together with the IRS, treated bed nets likely contributed to the reduction of malaria from May 2011 to October 2012, a period which witnessed the lowest slide positive rate (21.6% based on adjusted RDT findings) over the 6-year observation (Fig. 2). In late 2014, with a planned rotation, bendiocarb was replaced with an organophosphate compound (pirimiphos-methyl) to mitigate the development of resistance. The decisions to change insecticides were predicted on data provided by regular SMPS alerting the programme on control performance indicators including routine insecticide susceptibility testing of the local vector population.

Although a far greater majority students without fever were found infected with malaria (96%) than the few who presented with fever at time of examination, there was a significant association between those with fever (≥ 37.5 °C axillary temperature) and having a malaria infection (41.8%, p = 0.002) in both survey periods of the year. Students with fever were 1.3 times as likely to be infected with malaria compared to the group without fever. This agrees with other studies showing a strong linear relationship between the percentage of febrile children with malaria and infection prevalence [37, 38]. When data were aggregated by survey location (urban and rural), there was also a significant association between presence of fever and malaria infection in urban localities (62% of fevers had malaria) while showing no such relationship in rural areas (only ~ 30% of fevers had concurrent malaria). It is surmised that children from rural areas might have a greater risk of febrile illness due to other causes and/or possibly a higher level of naturally acquired immunity to repeat malaria infection thereby suppressing febrile episodes. It is also a possibility that rural children might be more likely not to attend school on day of fever regardless of reasons (i.e., due to other demands rather than feeling ill such as assisting family in the field). These possibilities would require further investigation to determine which factors might be responsible for separating -urban and rural child populations.

Different age groups among school-aged children have been used to estimate the risk of malaria infection in an area. From examples in Uganda [39], Malawi [40], Mozambique [41], Equatorial Guinea [40], Côte d’Ivoire [42], Senegal [43], Tanzania [44], school-age children used for surveys were between 5 and 9 years of age. Other studies conducted in children in Uganda, Kenya, Tanzania, Mali, Nigeria, Central African Republic, Cameroon, Congo Brazzaville, Ethiopia, Somalia, Yemen and Ghana have selected ages from 0.5 up to 18 years [19, 20, 45–50]. A systematic review on studies from Africa published in a 20-year period defined ‘school-age children’ as those between 5 and 14 years [5]. The ages of students sampled ranged from 6 to 12 years. The DRC Ministry of Education defines the age of 6 as the legal age to begin the first year of primary school [51]. It was unusual in the context of these surveys to have children completing all primary school grades before 12 years of age. Six to 12 years of age is in line with most mathematical models linking malaria infections (transmission) with age of students [6, 8].

Malaria infection was aggregated in three age groups: 6–8, 9–10 and 11–12 years of age. Overall, malaria prevalence in these age groups were 32.8, 35.4 and 34.0%, respectively. Malaria infection was independent of age or gender and there was no significant association between presence of malaria infection and age groups. In Kinshasa, Kazadi et al. [33] examined school-based malaria surveys conducted between 1981 and 1983 and found no significant difference in parasite rates by sex in any age group (5–9, 10–14 and ≥ 15); whereas infection prevalence increased significantly (p < 0.001) with age. The authors attributed this infection discrepancy to younger children being more prone to becoming more symptomatic with malaria and therefore less likely to attend school those days when ill compared to older children. Thus, confounding caused by increasing levels of naturally acquired immunity (i.e., decreased likelihood of more severe morbidity) in the older children enabled a higher probability of them attending school despite infection. They also surmised the difference might be that younger children received antimalarial treatment more often as was also described in Brazzaville [52] to explain why older children had an apparent higher malaria prevalence. Both Kinshasa and Brazzaville are large urbanized areas and show little resemblance to the more rural-based, smaller population of the Tenke-Fungurume area. In rural Ghana, Sarpong et al. [20] found a strong variation of malaria prevalence across schools, but a declining risk of parasitaemia (mean parasite densities) with age; whereas the frequency of patent infections without fever was higher in the more remote villages. The general decline in malaria incidence as age increases has been documented in other areas of Africa [53]. For example, the slide positivity rate (SPR) has been used to estimate changes in malaria incidence (number of cases per person-time) in Ugandan children aged 1–10 years and only those presenting with fever at time of testing [54]. They reported that younger age was associated with significantly greater risk of malaria and even more so during the seasonal peaks of transmission. Thus, it was concluded the SPR could be used as a surrogate measure in routine surveillance to describe changes in burden of malaria and incidence between seasonal variation and different age groups.

In this current 6-year retrospective analysis of school-based prevalence in DRC, no significant differences in malaria infection between age groups in the sampled population was seen. This may indicate that twice-yearly surveys may not be sensitive enough monitoring to detect a difference, if present. The SPR can be a useful measure of the impact of control interventions when considering several important caveats stressed by Jensen et al. [54]. First, a change in SPR does not necessarily equate to a proportional or linear change in actual incidence; second, age and seasonality can affect incidence and, therefore, may distort the actual impact of interventions and third, although the SPR can be used to estimate relative temporal-spatial changes in malaria incidence, it is not an estimate of actual incidence in the population. Unlike the Ugandan study, in the DRC analysis, children were randomly selected from the general population, regardless of fever status at time of sampling, thus the SPR was not directly affected by any change in incidence in non-malarial fevers that would potentially result in a change in SPR by selection bias and not reflective of true change in malaria incidence.

Findings showed no difference in malaria prevalence by gender. Other than apparent equal exposure to malaria risk as there would be no apparent occupational-related risk factor in most children at or below 12 years of age. With this younger age group, parents would be as likely to provide the same level of health care to offspring regardless of gender. Female gender inequality, when present, is generally observed into later adolescence and adulthood [55, 56].

In the SMPS, the overall risk of being infected by location was 1.92 greater in rural compared to urbanized localities, regardless of time of year. These results indicate a strong link between malaria transmission and location. A meta-analysis of entomologic inoculation rates (EIR) from urban, peri-urban and rural studies published between 1977 and 2000 reported a mean annual EIRs of 7.1 in city centers, 45.8 in peri-urban areas and 167.7 in rural areas of sub-Sahara African countries sampled [57]. Other studies made the similar conclusion that malaria is far more prevalent in rural compared to urban areas. In rural settings, the proximity to permanent larval habitats sites is probably the aggravating factor [58]. However, rural environments have also indirect links to increased malaria risk through several factors including lower health spending and more limited social-health resources [59]. In urban settings, although malaria transmission could be low due to the urbanization/environmental changes itself, other factors like pollution could adversely affect the suitability of larval habitats, mosquito survival and ability or likelihood to transmit malaria (i.e., vectorial capacity). Other differences might include better housing with physical barriers such as screens, doors, use of insecticides and bed nets. Higher human population densities may also reduce individual biting rates (thus risk), owing to the higher ratio of humans to mosquitoes [60, 61].

The distribution of malaria infections was significantly different between rural and urban HAs and between May and October surveys, thus indicating an interaction between time of year and location. In May 2012, while the overall malaria prevalence in the concession decreased, there was a significant increase in malaria in rural localities and a decrease in urban localities. This suggested that additional interventions to the existing methods to control malaria (IRS and LLINs) were required in rural localities to reduce the malaria burden. In response, a pilot study on community-based malaria diagnostic and treatment was conducted from 2012 to 2013 in 14 rural villages. Results indicated that a scaled-up community-based approach using trained and supervised community health workers could be an effective and promising additional strategy to control malaria and an addition to the integrated MVCP [62].

The prevalence of single infections seen with *P. falciparum*, *P. malariae* and *P. ovale* were 29.9, 1.8 and 0.3%, respectively. *P. malariae* and *P. ovale* where consistently detected at low rates compared to *P. falciparum*, and typically at low density. In another study, Congolese children under the age of five showed a higher prevalence of *P. malariae* and *P. ovale*, 12.9 and 8.3%, respectively [63]. The variation is likely attributed to the difference of the age groups sampled (under 5 versus 6–12 years of age in this 6-year TFM retrospective analysis) as well as the diagnostic methods used (PCR versus expert microscopy) [34].

Overall, there was a significant association, albeit weak, between *P. falciparum* density and different age groups (*p *= 0.039). Additionally, there was an inverse correlation between survey period and *P. falciparum* densities. However, in rural localities, the correlation was not different to zero while it was significantly different in urban localities, i.e. during intervention the mean *P. falciparum* density decreased over time in urban localities while showing no changes in rural localities.

Lateral flow immunochromatographic parasite protein-detecting assays (known more commonly as rapid diagnostic tests) can provide generally accurate diagnosis for case management, often with a similar analytical accuracy to microscopy when using a high-quality product, in good condition, and prepared and interpreted correctly by a trained operator. RDTs are more amenable for use in remote locations and less dependent on higher skill levels to perform [64]. Although RDTs are a major advancement to malaria control, there are several limitations present in the current products on the market and depending on test detection format. For example, one of the most significant drawbacks is reduced test sensitivity when parasite densities are low [65], compared to other methods (e.g., expert microscopy, PCR). Therefore, to enable capturing a greater number of lower parasite density infections in our sampled population, we took the additional step of having matched blood films carefully examined by expert microscopy.

Typically, *Pf*HRP2-detecting RDTs outperform (better sensitivity) than non-*Pf*HRP2 assays that use either aldolase or *Plasmodium* lactate dehydrogenase (*p*LDH) enzymes for detecting infections. As *P. falciparum* is overwhelmingly the predominant malaria parasite species seen in the FHZ, we deliberately selected high quality *Pf*HRP2 detection RDTs. Greater thermal stability compared to *p*LDH-based RDTs and product availability also influenced the choice of product [66].

The recognition of *Pf*HRP2 and *Pf*HRP3 parasite gene deletions that can result in false negative RDTs [67] should be assessed at TFM site. Currently, based on the reported low frequency of this genetic anomaly in Africa and the low percentage of false negative HRP2 RDT results identified in this observation with infections showing moderate to high density *P. falciparum* parasitaemias, this is not view as a substantial issue, if any, in the FHZ at this time. If later deemed a problem, it will raise the need for using tests that use non-HRP2 *falciparum*-specific targets, i.e., pLDH, either pan (all *Plasmodium* species) or *P. falciparum*-specific, alone or in combination with HRP2. In future, those cases presenting with high parasitaemias that produce negative RDT results could be tested for possible described gene deletions to explain false negative results. Additionally, hyper-parasitaemia can result in an apparent ‘prozone’ effect due to an antigen overload resulting in a false negative result [68]. Prozone is also regarded an infrequent occurrence but appears a common limitation to many HRP2-based RDTs at frequencies that may diminish diagnostic accuracy. Conversely, *Pf*HRP2 has been shown to persist and can be detectable many days, even weeks after the clinical symptoms of malaria have disappeared and the parasites have apparently been cleared from the host [69]. This would lead to a lower RDT specificity (false positive signal). However, low-level parasitaemias more common areas of constant exposure to malaria parasites may result in positive findings due to persistent circulating HRP2 from previously cleared parasitaemias and not necessarily because of a current infection. Moreover, one other limitation to HRP2-based RDTs is the weak relationship between HRP2 quantitative blood level and the peripheral parasite density [65]. HRP2-based RDTs do not detect the parasites per se but rather the concentration of protein analyte in circulation. Thus, it is not possible to translate amounts of target antigen produced by parasites into percentage of parasitized red blood cells or parasites per µl of blood, two commonly used thresholds describing the degree of infection (i.e., severity). Therefore, comparing RDT performance sensitivity with microscopy results can present interpretive hurdles. Nevertheless, the addition step of using light microscopy to quantitatively measure parasite densities provided valuable information on malaria in the intervention area that would not be available using an RDT alone.

In the school-aged population surveyed, the RDT sensitivity (false negative) has been a notable limitation as the vast majority of such cases appear to have been the result of low-level parasitaemias/asymptomatic infections (generally densities below 100 asexual parasites/µl peripheral blood). This would be of less clinical significance compared to having greater epidemiological implications as repeat infections due to regular exposure are more likely to become subclinical parasite reservoirs.

Many contributing factors and limitations in data collection are acknowledged in the SMPS presented, but many were beyond the scope, practicality, and intention of this routine monitoring exercise. For example, time and resources were not available to follow-up registered children not attending school the day of sampling. Children could have been absent for many reasons, one possibility being illness caused by malaria. The loss of children with malaria would have biased the findings and analysis on factors such as age and fever. However, to what extent is not known. Different school’s attendance rolls the day of survey were not compared to determine percent absence and if differences were significant between locations (rural vs. urban, for example). Depending on location, local demographics may vary considerably, thus a contributing for likelihood of school attendance. For example, in rural areas, more dependent on subsistence seasonal agriculture, a child’s absence may be the result of having to assist in the field during peak planting or harvesting periods. On the other hand, school absenteeism in the urban area could be due to greater access or more frequent travel outside the area. Lastly, it would have been preferred that two persons independently examine the blood films using light microscopy. However, this was deemed prohibitively expensive and time consuming. Moreover, no matter the number of independent reads, some very low density/sub-patent infections will go undetected nevertheless. Given the level of expertise of the microscopist used for all 11 surveys, the authors are confident the results are accurate enough and comparable across surveys for the intended purposes described herein. Lastly, fever (≥ 37.5 °C) was measured using a digital device placed in the axillary area. Axillary temperatures are usually 0.3–0.6 °C lower than an oral temperature. An axillary temperature is influenced by the temperature on the outer surface of the body. A normal axillary temperature is between 35.9 and 36.7 °C. In other words, ‘fever’ during sampling was likely underestimated. While oral readings are typically more accurate, they can take longer to measure than axillary temperatures and thus impede mass sampling efforts. Although the overall absolute number of fevers may have been lower using the axillary method, as all children were measured in the same way, this should have had no meaningful impact on the statistical comparisons.

The surveys and findings were solely derived for operational purposes, not as controlled experiments, and thus output has some inherent constraints. The school malaria observations were made as part of an evidenced-based monitoring strategy to provide important metrics (i.e., disease prevalence) to directly assist the control programme. What is presented is the first known longitudinal set of data on malaria prevalence in school children in a defined, but rapidly expanding, population in southern DRC. To this point, recent information on the malaria burden in rural areas of the Katanga region (formerly Katanga Province, now sub-divided into four separate provinces), has remained fragmented, anecdotal, or non-existent in most instances. Since the cessation of organized malaria control programmes in the area in the 1980s mainly by state-sponsored companies such as the mining operations (*Gécamines*) and the DRC Railway Company (*Société Nationale de Chemin de fer du Congo*-*SNCC*), there has remained a large gap in basic epidemiological data and information on malaria in the region [70].

Effective malaria control and planning requires accurate measurements and information on both the geographical distribution of malaria risk and the effectiveness of malaria interventions [16, 71–73]. School-based surveys of children can provide a rapid, sensitive, convenient, and sustainable approach to malaria disease monitoring and evaluation and have been recommended as a complement to passive heath care statistics and household surveys [4, 5, 16, 19, 20, 74]. By contrast, household surveys, although informative, are generally more expensive, time and labor intensive and are more typically undertaken every 3–5 years or at longer time intervals [16, 74]. Advantageously, school-based surveys can be conducted more frequently, while also concentrating on an age group more likely to reside in the area of interest the majority of the time. Unlike older adolescents and adults who may travel out of the area more frequently and thus have a different malaria exposure risk profile. Pre-school children (< 60 months) would also be more likely to represent local exposure, however this age group poses two distinct disadvantages—typically a less convenient cohort to access that often requires door-to-door visits, and lower sample representation in a population. In the FHZ, the biannual SMPS will continue to be performed to monitor impact of the malaria control programme, the effects of interventions by location and time. This approach has provided important operational performance indicators to assist in programme decisions on control prioritization, use of insecticides, and timing of interventions. In operational control programme settings, longitudinal epidemiological measures are needed to measure the changing landscape of malaria risk over time. Better utilization of school-based health surveys (not just malaria) in ongoing surveillance programmes, support of health management information systems, and identifying most prudent use of current control methodologies should be promoted.

## Conclusion

The school-based malaria prevalence survey is a valuable evidence-based estimate for observing changes in the intensity of malaria and assess the impact of malaria control interventions. It can assist in identifying areas requiring additional priority interventions and provides critical information for programme orientation. When well executed and where school participation rates are high, prevalence surveys should be considered a routine and valid malariometric tool to measure malaria transmission in most endemic areas.

## Abbreviations

ACT: artemisinin-based combination treatment
DRC: Democratic Republic of the Congo
FHZ: Fungurume Health Zone
HA: Health Area
IRS: indoor residual spray
LLINs: long-lasting insecticide treated bed nets
MVCP: malaria and vector control programme
NMCP: National Malaria Control Programme
PR: prevalence ratio
SMPS: school-based malaria prevalence survey
TFM: Tenke Fungurume Mining
WHO: World Health Organization
